# *In situ* fabric-integrated cellulose hydrogels *via* one-step crosslinking for silver-based wound dressings

**DOI:** 10.1039/d6ra03102a

**Published:** 2026-07-13

**Authors:** Farooq Azam, Saif Ullah, Faheem Ahmad, Rashid Masood, Sheraz Ahmad, Bushra Mushtaq, Abher Rasheed, Sehar Sajid, Muhammad Sohail Zafar

**Affiliations:** a School of Engineering and Technology, National Textile University Faisalabad Pakistan sheraz@ntu.edu.pk faheem@ntu.edu.pk; b Department of Clinical Sciences, College of Dentistry, Ajman University Ajman United Arab Emirates; c Centre of Medical and Bio-allied Health Sciences Research, Ajman University Ajman United Arab Emirates; d School of Dentistry, University of Jordan Amman Jordan; e Department of Chemistry, University of Agriculture Faisalabad Pakistan

## Abstract

This study presents the development of a hydrogel-based wound dressing by incorporating carboxymethyl cellulose and silver nanoparticles into cotton fabric *via* a one-step method. The objective was to create a cellulose-based wound care material that offers good strength, comfort, moisture management, and antibacterial protection, using an industrially viable, simple approach. Experimental evaluations assessed mechanical strength, stretchability, moisture management behavior, water absorption capacity, and antibacterial activity. Results showed that the material's strength increased with greater amounts of hydrogel and fabric weight. The highest tensile strength of 120 N and the lowest elongation of 52% were achieved with 1.25% hydrogel and a fabric weight of 150 g m^−2^. The dressing demonstrated excellent comfort properties, including a fast-wetting time of 1.73 s and a high liquid spreading rate of 17 mm s^−1^, which are beneficial for wound healing. Moreover, greater hydrogel content and fabric weight enhanced fluid absorption, making the material suitable for managing wound exudate. The inclusion of silver nanoparticles significantly inhibited bacterial growth around the dressing, confirming its antimicrobial effectiveness. These findings highlight the potential of the newly developed one-step approach for cellulose hydrogel-coated cotton fabric as a multifunctional, effective wound dressing.

## Abstract 2

A one-step *in situ* crosslinking strategy integrates cellulose hydrogel and silver nanoparticles into cotton fabric, producing a multifunctional wound dressing with enhanced strength, moisture management, absorbency, and antibacterial activity.

## Graphical abstract

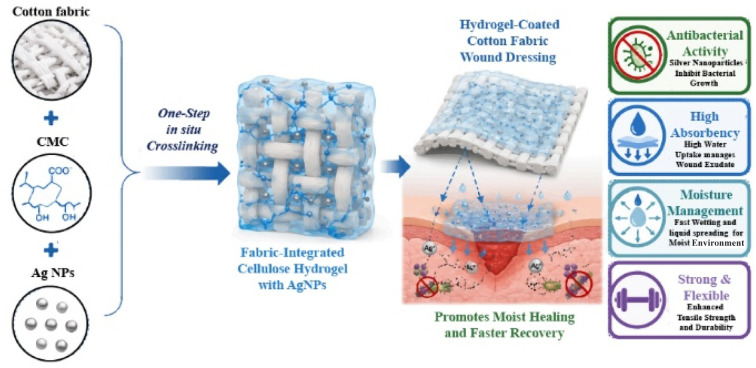

## 1 Introduction

Cellulose and its derivatives have been widely investigated for biomedical applications due to their biocompatibility, hydrophilicity, and biodegradability.^1–3^ Among these, carboxymethyl cellulose (CMC), a water-soluble cellulose derivative, has been extensively studied for hydrogel formation because of its numerous functional groups (carboxyl and hydroxyl) that facilitate network formation and water retention. Hydrogel networks can be formed *via* covalent, ionic, or physical crosslinking mechanisms. Covalent crosslinking involves the formation of permanent chemical bonds between polymer chains.^4^ Typical covalent crosslinkers include small multifunctional molecules such as citric acid (which esterifies hydroxyl groups to form stable networks), glutaraldehyde, and epichlorohydrin.^5^ Citric acid has been widely used to create CMC hydrogels and hybrid CMC–PVA networks with tailored swelling behavior and mechanical properties for biomedical use.^6^ Physical crosslinking does not form new covalent bonds but relies on hydrogen bonding, van der Waals interactions, or chain entanglement to form a hydrogel network. These networks are often reversible and easily disrupted in aqueous environments, which can limit long-term stability.^7,8^ Ionic crosslinking uses multivalent metal ions to form junction points *via* electrostatic interactions with anionic sites (*e.g.*, carboxylate groups in CMC). While this approach has been explored for other polysaccharides, such as alginate, its use in CMC hydrogels for biomedical applications, particularly wound dressings, is less common in the literature.^9^

Hydrogel-based wound dressings are highly competitive due to their drug delivery and extracellular matrix (ECM) mimicking properties.^10,11^ These hydrophilic polymers form swellable, non-soluble 3-D networks capable of absorbing and retaining large amounts of fluid, providing pain relief, regulating temperature, and allowing oxygen transmission to enhance healing.^12^ Natural polymers like hyaluronic acid, alginate, cellulose, chitosan, and collagen are commonly used.^13,14^ Among various hydrogel materials, cellulose and its derivatives, particularly carboxymethyl cellulose (CMC), have emerged as highly promising candidates owing to their biocompatibility, biodegradability, non-toxicity, hydrophilicity, and excellent swelling properties. Many studies focus on CMC blended with other polymers like gelatin, and physical crosslinking approaches have been investigated for enhanced wound healing performance.^15^

Cellulose-based hydrogels lack inherent antimicrobial activity, making them susceptible to bacterial colonization when applied to infected wounds.^3^ Moreover, antibacterial strategies often involve incorporating nanoparticles or antimicrobial agents,^16^ such as silver (Ag) or zinc oxide, into CMC matrices to control wound infections.^17,18^ To address this limitation, silver nanoparticles (AgNPs) have been widely incorporated into wound dressings due to their broad-spectrum antibacterial activity,^19^ long-term effectiveness, and low tendency toward microbial resistance.^2,20^ Pure hydrogels are often brittle, mechanically weak, and prone to structural failure even under minor applied forces.^21^ Therefore, numerous studies have focused on improving the mechanical performance of hydrogels by reinforcing them with textile substrates, such as nonwoven fabrics.^22–24^ This reinforcement strategy enhances the strength, stability, and handling properties of hydrogels while improving their water-absorption capacity, making them more suitable for practical wound-dressing applications.^25,26^ Cotton nonwoven fabric is an affordable, biodegradable option that supports sustainable production with minimal environmental impact. Research indicates that a moist environment around the wound facilitates swift healing, making CMC-based hydrogels ideal due to their excellent absorbency and hydrophilic properties.^27^ Additionally, the biodegradable nature of cellulose-based hydrogels contributes to environmental sustainability.

Despite the extensive body of work on covalently crosslinked and blended CMC hydrogels, hydrogels formed *via* multivalent metal-ion-induced ionic crosslinking, particularly on textile supports, have received limited attention in the wound-dressing literature. Traditional ionic crosslinking is widely used for polysaccharides such as alginate, where Ca^2+^ or other ions form egg-box networks, but similar strategies for CMC directly on fiber substrates (*e.g.*, cotton nonwoven) are less represented. Therefore, developing a simple ionic crosslinking strategy that forms CMC hydrogel networks directly on cotton nonwoven fabric, followed by functionalization with antimicrobial nanoparticles, could offer a novel pathway for advanced wound dressings that combine mechanical support, fluid management, and infection control. This study addresses this by developing a cotton-reinforced cellulose hydrogel composite using a novel, one-step process that is both simple and commercially viable. The hydrogel is applied to non-woven fabrics with different GSM values to create the dressing. The resulting composites are thoroughly characterized for absorbency, mechanical strength, and chemical structure using FTIR. Furthermore, AgNPs are incorporated to impart enhanced antibacterial properties. The performance of these dressings is evaluated to determine the optimal formulation for wound care applications.

## 2 Materials and methods

### 2.1 Materials

CMC powder with 0.8 degree of substitution and a molecular weight of 600 kDa, sourced from Daejung Korea, was used as the precursor for the hydrogel. Silver nitrate (AgNO_3_) from Sigma-Aldrich served as the precursor for synthesizing silver nanoparticles. Dimethyl sulfoxide (DMSO) and aluminum sulfate, both from Sigma-Aldrich, served as the reducing and cross-linking agents, respectively.

### 2.2 Design of experiment

Table 1 presents the hydrogel solution concentrations and the corresponding grams per square meter (GSM) ranges.

**Table 1:** Design of experiment

Sr. no.	Hydrogel solution %	Nonwoven fabric GSM
1	0.75	150
2		100
3		50
4	1	150
5		100
6		50
7	1.25	150
8		100
9		50

### 2.3 Method

#### 2.3.1 Preparation for hydrogel solution

Five hundred milliliters of distilled water were measured for each hydrogel solution concentration. CMC powder was added gradually to the distilled water according to the DOE while it was continuously stirred. The mixture was stirred vigorously until the CMC powder was uniformly dispersed, using a magnetic stirrer set at 800 rpm. Stirring was conducted until a homogeneous solution was formed, resulting in the preparation of CMC hydrogel solutions in different beakers.

#### 2.3.2 Preparation of silver nanoparticles

Silver nanoparticles (AgNPs) were prepared by first dissolving 0.5 grams of silver nitrate in 100 mL of distilled water. This solution was stirred vigorously for 30 minutes. Subsequently, 100 mL of dimethyl sulfoxide (DMSO) was added to the mixture, and the reaction was stirred continuously for approximately 3 hours to facilitate nanoparticle formation.

#### 2.3.3 Application of hydrogel and silver nanoparticles

**Fig. 1:**
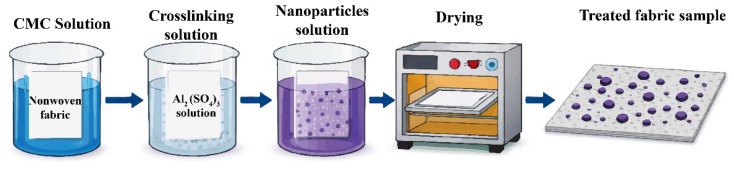
Schematic diagram to develop the hydrogel composite sample.

Nonwoven fabric sheets were immersed in the prepared CMC hydrogel solutions at room temperature to ensure uniform coating of the fabric surface. Sheets with different GSM values were dipped in their respective hydrogel formulations for 30 min at room temperature. Excess hydrogel was removed using a laboratory padder to obtain a uniform wet pick-up. The hydrogel-coated sheets were subsequently immersed in an aqueous aluminum sulfate (Al_2_(SO_4_)_3_) solution (0.5 molar) using a liquor ratio of 1 : 20 (fabric : solution, w/v) for 30 min at 25 ± 2 °C to facilitate ionic crosslinking of the CMC hydrogel network. After crosslinking, the samples were rinsed lightly at room temperature with distilled water to remove any loosely bound crosslinking agent. The crosslinked sheets were then immersed in the prepared nanoparticle dispersion (0.5 w/v%) for 60 min at room temperature to allow nanoparticle diffusion and incorporation into the hydrogel-coated nonwoven structure. Finally, the samples were dried in a hot-air oven at 40 °C for 20 min. Fig. 1 illustrates the fabrication procedure of the hydrogel composite samples.

### 2.4 Characterization

#### 2.4.1 Surface morphology

A light microscope was employed to examine the surface morphology of the hydrogel composite samples. This technique enabled visual assessment of fiber-hydrogel integration, surface smoothness, and any irregularities or defects that may have formed during fabrication.

#### 2.4.2 Fourier transform infrared spectroscopy (FT-IR)

Fourier Transform Infrared (FTIR) Spectroscopy was performed using a Jasco FTIR-6x model over the 600–4000 cm^−1^ range to confirm functional groups and verify the successful incorporation of a carboxymethyl cellulose (CMC)-based hydrogel into the nonwoven cotton bandage. Since the samples were in solid state, the process began by placing each sample on the crystal's surface. Next, a gripper plate was placed on top of the sample, and the pressure applied was adjusted to ensure consistent contact between the crystal and the sample.

#### 2.4.3 Mechanical properties analysis

Elongation and tensile strength are determined using the ISO 13934-2:2013 testing method.

#### 2.4.4 Zeta sizer analysis

Malvern ZetaSizer equipment is used to measure the size of silver nanoparticles in nm that exist on the non-woven hydrogel fabric.

#### 2.4.5 Moisture management

The SDL-ATLAS moisture management tester (MMT) is used for moisture management in accordance with the AATCC 195 standard test protocol. With this method, a 3 × 3 cm cloth surface is wetted with 0.91% saline solution, and the moisture properties are measured using MMT.

#### 2.4.6 Exudate absorbency

The ability of the nonwoven cotton and CMC-based cotton hydrogel composite to absorb wound exudate was evaluated in accordance with the standard EN 13726-1:2002.

#### 2.4.7 Antibacterial property

Using the “Agar disk diffusion” method, antibacterial activity is determined. The American Type Culture Collection No. 6538, the Gram-positive bacterium *Staphylococcus aureus*, was examined for its antibacterial properties. The material was sterilized in steam at 121 °C for 15 minutes before testing. The substance was then applied to the agar disk and incubated for 18 hours. The antibacterial activity of the samples was observed.

## 3 Results and discussion

### 3.1 Surface morphology

**Fig. 2:**
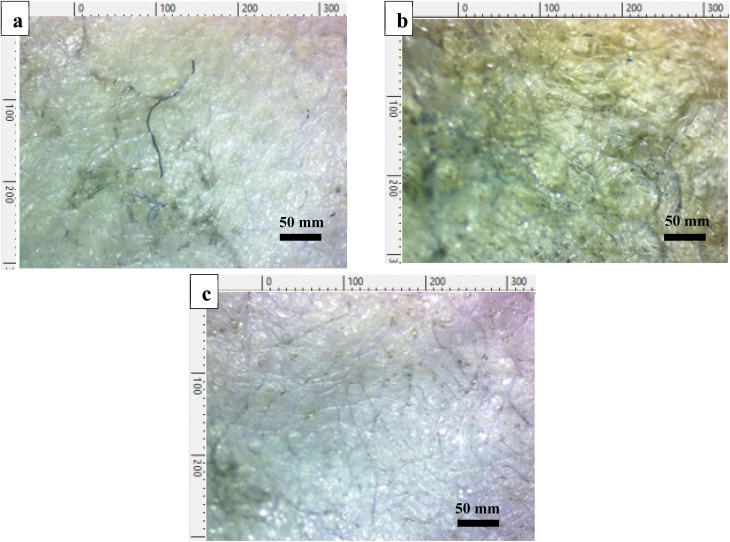
Light microscope images of hydrogel samples with 150 GSM at (a) 0.75% concentration, (b) 1% concentration, and (c) 1.25% concentration.

Fig. 2 displays microscopy images of samples at 10× magnification, showing cellulose hydrogel concentrations of 0.75%, 1%, and 1.25%, labeled as 1, 4, and 7, respectively. The cellulose hydrogel matrix appears as a network of interconnected fibers, forming a continuous phase. This matrix exhibits a porous structure, indicating the presence of interstitial spaces within the network that can absorb fluids, a key feature for wound-healing applications. The images also reveal silver nanoparticles as small, dark spots scattered throughout the hydrogel matrix. Their distribution appears generally uneven, likely due to the hand-layup method used for application.

### 3.2 Fourier transform infrared spectroscopy (FTIR)

**Fig. 3:**
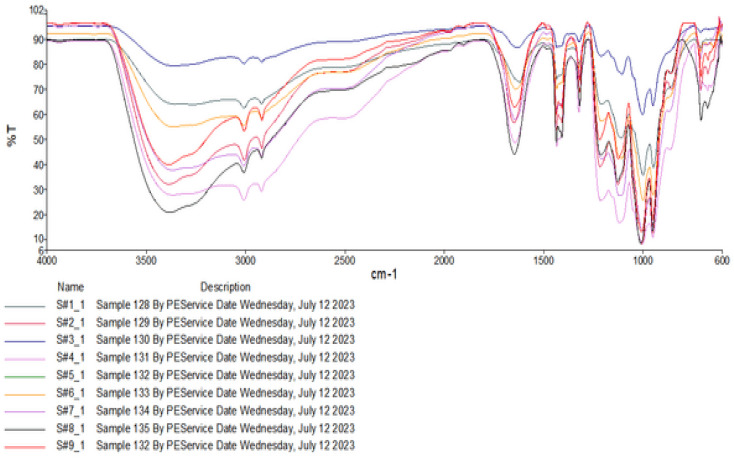
FT-IR analysis of all the samples.

The Fourier Transform Infrared (FTIR) spectra of the non-woven cotton bandage samples are presented in Fig. 3. All samples exhibit absorption bands at identical wavenumbers, indicating consistent fabrication of the hydrogel-treated bandages across GSM and hydrogel concentration. The broad absorption band centered around 3450 cm^−1^ is attributed to the stretching vibration of hydroxyl (–OH) groups, originating from both the cotton cellulose backbone and the CMC-based hydrogel. The peak observed at approximately 2900 cm^−1^ corresponds to C–H stretching vibrations of aliphatic groups in cellulose.

Although the characteristic peaks appear at the same wavenumbers across all samples, noticeable variations in peak intensity are observed, which can be ascribed to differences in non-woven GSM and hydrogel loading. Samples 1, 4, and 7 exhibit higher peak intensities, which are consistent with their higher fabric GSM (150 GSM) and corresponding hydrogel concentration, resulting in increased hydroxyl and polymeric content within the composite structure. Furthermore, the FTIR spectra display distinct absorption bands at 1618, 1423, and 1061 cm^−1^. The band around 1618 cm^−1^ is associated with the asymmetric stretching of carboxylate (–COO^−^) groups present in the CMC hydrogel, while the peak at 1423 cm^−1^ corresponds to symmetric carboxylate stretching. The strong absorption at 1061 cm^−1^ is attributed to C–O–C stretching vibrations of the cellulose and CMC backbone. In addition, the absorption band observed at approximately 602 cm^−1^ in the CMC hydrogel is attributed to carboxyl functional groups.

The presence of these characteristic peaks, along with their enhanced intensities after hydrogel treatment, confirms the successful incorporation of the CMC hydrogel and suggests strong interfacial interactions—primarily hydrogen bonding and ionic interactions—between the cotton fibers and the hydrogel matrix. Moreover, the retention of all major cellulose peaks indicates that the cotton's fundamental chemical structure remains intact after treatment. Overall, the FTIR results demonstrate effective adhesion between the silver nanoparticle-loaded CMC hydrogel and the cotton bandage surface, thereby confirming the successful formation of the composite wound dressing material.

### 3.3 Tensile strength

**Fig. 4:**
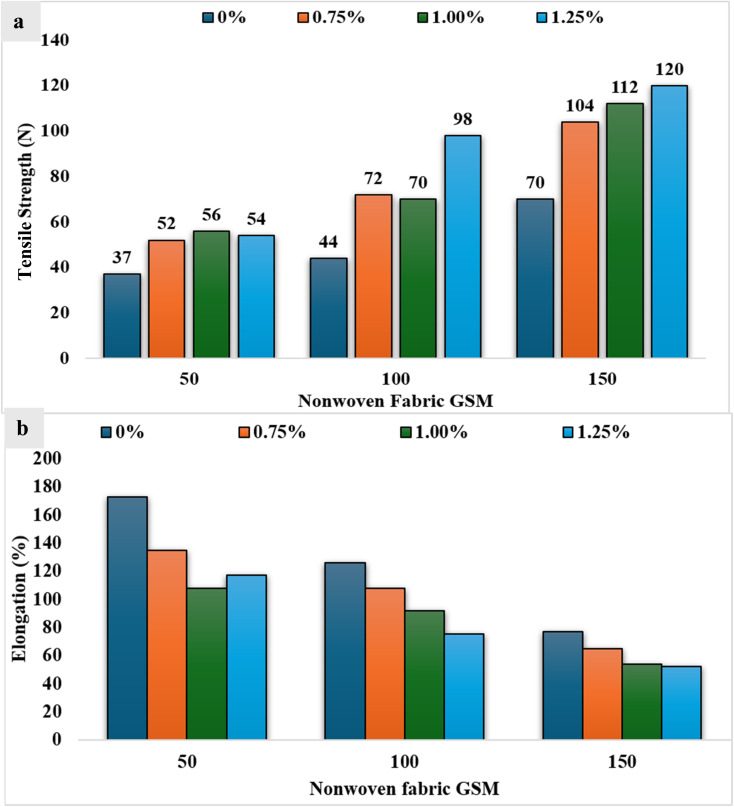
Effect of GSM and hydrogel concentration on (a) tensile strength and (b) elongation at break of hydrogel samples.

The tensile strength of the samples was characterized across different hydrogel concentrations and fabric GSM, as shown in Fig. 4a. For the samples without hydrogel, the tensile strength decreased with decreasing GSM: 70 N at 150 GSM, 44 N at 100 GSM, and 37 N at 50 GSM. When 0.75% CMC hydrogel was applied, the tensile strength improved to 104 N, 72 N, and 52 N for the respective GSM. Further enhancement was observed with 1% CMC hydrogel, achieving tensile strengths of 112 N, 70 N, and 56 N for 150, 100, and 50 GSM, respectively. The highest tensile strength was observed with a 1.25% CMC hydrogel concentration, reaching 120 N at 150 GSM, 98 N at 100 GSM, and 54 N at 50 GSM. The observed increase in tensile strength with higher hydrogel concentrations and GSM of the fabric is attributed to several factors. Higher concentrations of carboxymethyl cellulose (CMC) hydrogel enhance the crosslinking and bonding between the hydrogel matrix and the cotton nonwoven fabric, resulting in a stronger composite structure. The integration of silver nanoparticles further reinforces this matrix, thereby improving mechanical stability. Fabrics with higher GSM provide a denser, more robust base, enabling better adhesion and mechanical support from hydrogel. The combination of these effects creates a dual-reinforcement mechanism: the CMC hydrogel provides structural support, and the silver nanoparticles add mechanical reinforcement, resulting in a significant improvement in tensile strength in the composite samples.

### 3.4 Elongation at break

The elongation results in Fig. 4b for the hydrogel composites show a clear trend of decreasing elongation percentage with increasing hydrogel concentration and fabric GSM. The pure cotton nonwoven samples (without hydrogel) exhibit the highest elongation percentages, with the lowest GSM (50) fabric demonstrating the greatest elongation at 173%. This suggests that the base cotton fabric, without hydrogel reinforcement, is more flexible and can stretch more. As the concentration of the CMC hydrogel increases, the elongation percentage decreases across all fabric GSM levels. For example, at a hydrogel concentration of 1.25, the elongation percentages are significantly lower, particularly for the highest GSM (150) fabric, which shows only 52% elongation. This reduction in elongation can be attributed to the increased rigidity imparted by the hydrogel matrix, which limits the fabric's flexibility. The addition of hydrogel stiffens the composite by filling the nonwoven fabric's pores and binding the fibers more rigidly, thereby reducing the material's ability to elongate. Furthermore, as the fabric GSM increases, elongation percentages tend to decrease. This trend indicates that denser fabrics, which contain more material per unit area, inherently possess less flexibility and stretchability. When combined with the CMC hydrogel, this rigidity is further enhanced, resulting in even lower elongation. The incorporation of CMC hydrogel into the cotton nonwoven fabric reduces the composite's elongation, with higher hydrogel concentrations and fabric GSMs resulting in stiffer, less stretchable composites.

### 3.5 Zeta sizer analysis

**Fig. 5:**
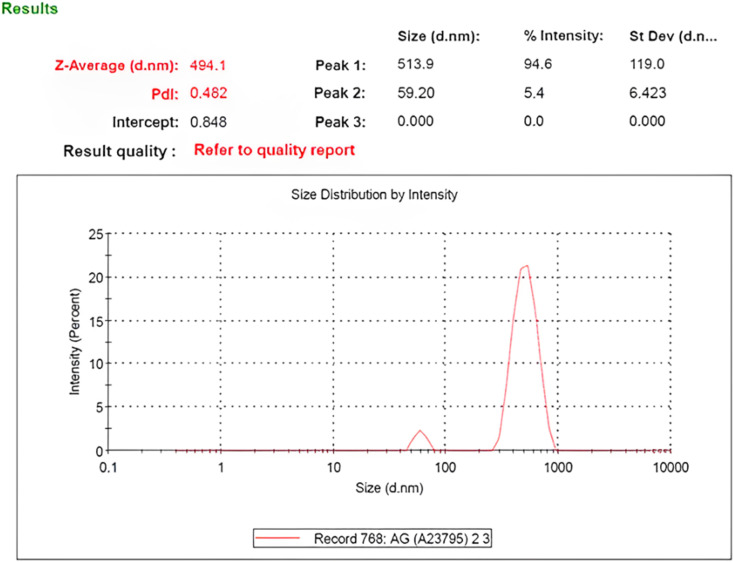
Particle size analysis of AgNPs after their incorporation into the hydrogel matrix.

Fig. 5 illustrates the experimental conditions, which included maintaining a temperature of 25 °C and ensuring that no material was absorbed. The *Z*-average (*D* nm) of the sample, representing the average particle diameter, was recorded at 494.1 nm. The intensity distribution reveals three distinct peaks. Peak 1 corresponds to particles with a size of 513.9 nm, an intensity of 94.6, and a standard deviation of 119 nm, indicating a predominant size distribution around 513.9 nm. Peak 2 shows particles of 59.20 nm in size, with an intensity of 5.4 and a standard deviation of 6.423, reflecting minor size fluctuations and a lower concentration than Peak 1. This shows that silver particles are of varying sizes.

### 3.6 Moisture management

The moisture management results for the hydrogel composite samples, reinforced with nonwoven cotton and containing silver nanoparticles, shown in Table 2, demonstrate significant variations with hydrogel concentration and fabric GSM. The wetting time for both the top and bottom surfaces decreases as hydrogel concentration increases, indicating faster moisture absorption. Specifically, samples with a 1.25% hydrogel concentration exhibit the shortest wetting times, with values ranging from 3.1 seconds on the top surface to 2.74 seconds on the bottom for the 150 GSM fabric.

**Table 2:** Moisture management results of all the samples

Sr. #	Hydrogel concentration	Fabric GSM (%)	Wet time top (sec)	Wet time bottom (sec)	Top spreading speed (mm s^−1^)	Bottom spreading speed (mm s^−1^)
1	0.75	150	2.28	2.24	16.57	3.48
2	0.75	100	1.98	2.09	15.09	2.98
3	0.75	50	1.23	1.12	8.98	1.39
4	1	150	2.85	2.49	16.82	3.73
5	1	100	2.23	2.34	15.34	3.32
6	1	50	1.48	1.37	9.23	1.64
7	1.25	150	3.1	2.74	17.07	3.98
8	1.25	100	2.48	2.59	15.59	3.57
9	1.25	50	1.73	1.62	9.48	1.89
11	0	150	67.09	17.42	0.17	0.3
12	0	100	62.05	15.03	0.16	0.26
13	0	50	22.5	6.5	0.98	0.18

The spreading speed also increases with higher hydrogel concentrations. For instance, the top spreading speed reaches 17.07 mm s^−1^ at a 1.25% hydrogel concentration on 150 GSM fabric, while the bottom spreading speed is 3.98 mm s^−1^, indicating enhanced moisture-spreading capabilities. In contrast, the control samples (0% hydrogel) exhibit significantly longer wetting times and much lower spreading speeds, indicating poor moisture management. This trend is consistent across all GSM levels, with lower-GSM fabrics generally exhibiting shorter wetting times and slower spreading rates than higher-GSM fabrics. The results suggest that increasing the hydrogel concentration in the composite significantly enhances the fabric's moisture management properties, making them more effective for applications requiring rapid moisture absorption and distribution.

### 3.7 Exudate absorbency property

**Fig. 6:**
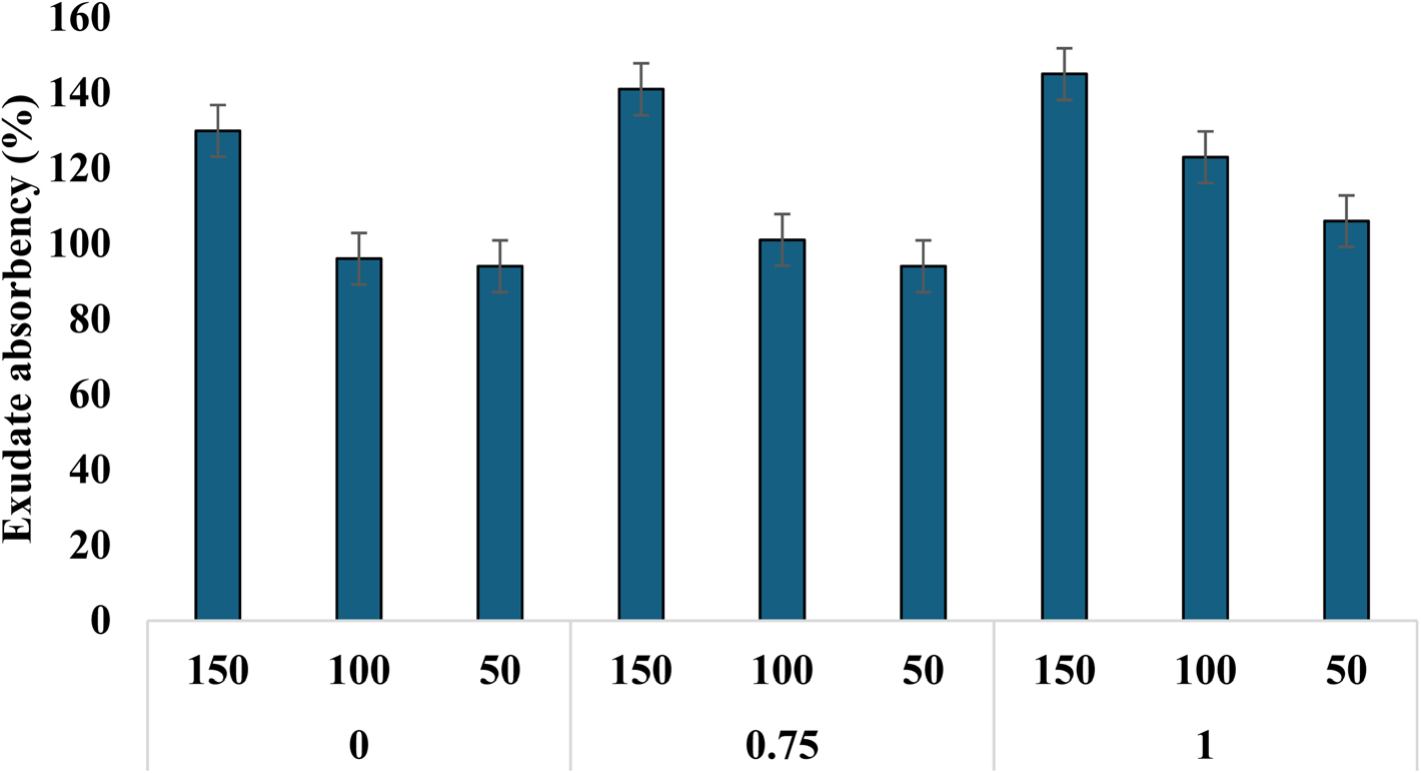
Exudate absorption of the hydrogel composite samples.

The exudate absorbency results for the hydrogel composite samples are shown in Fig. 6, which includes cotton nonwoven reinforced with carboxymethyl cellulose hydrogel and silver nanoparticles. The results indicate that both hydrogel concentration and fabric GSM (grams per square meter) significantly influence the absorbency performance. As the hydrogel concentration increases from 0.75% to 1.25%, exudate absorbency increases across all fabric GSM levels. For example, at a 0.75% hydrogel concentration, the exudate absorbency for 150 GSM fabric is 130%, while at a 1.25% hydrogel concentration, it rises to 145%. Similarly, for 100 GSM fabric, the absorbency increases from 96% to 123%, and for 50 GSM fabric, it goes from 94% to 106%. These results suggest that higher hydrogel concentrations enhance the material's ability to absorb exudates, likely due to the hydrogel's increased hydrophilicity. Additionally, the data indicate that higher GSM fabrics generally exhibit greater absorbency, which may be due to their increased thickness and capacity to hold more hydrogel, thus improving the composite's overall exudate management properties.

### 3.8 Antibacterial property results

**Fig. 7:**
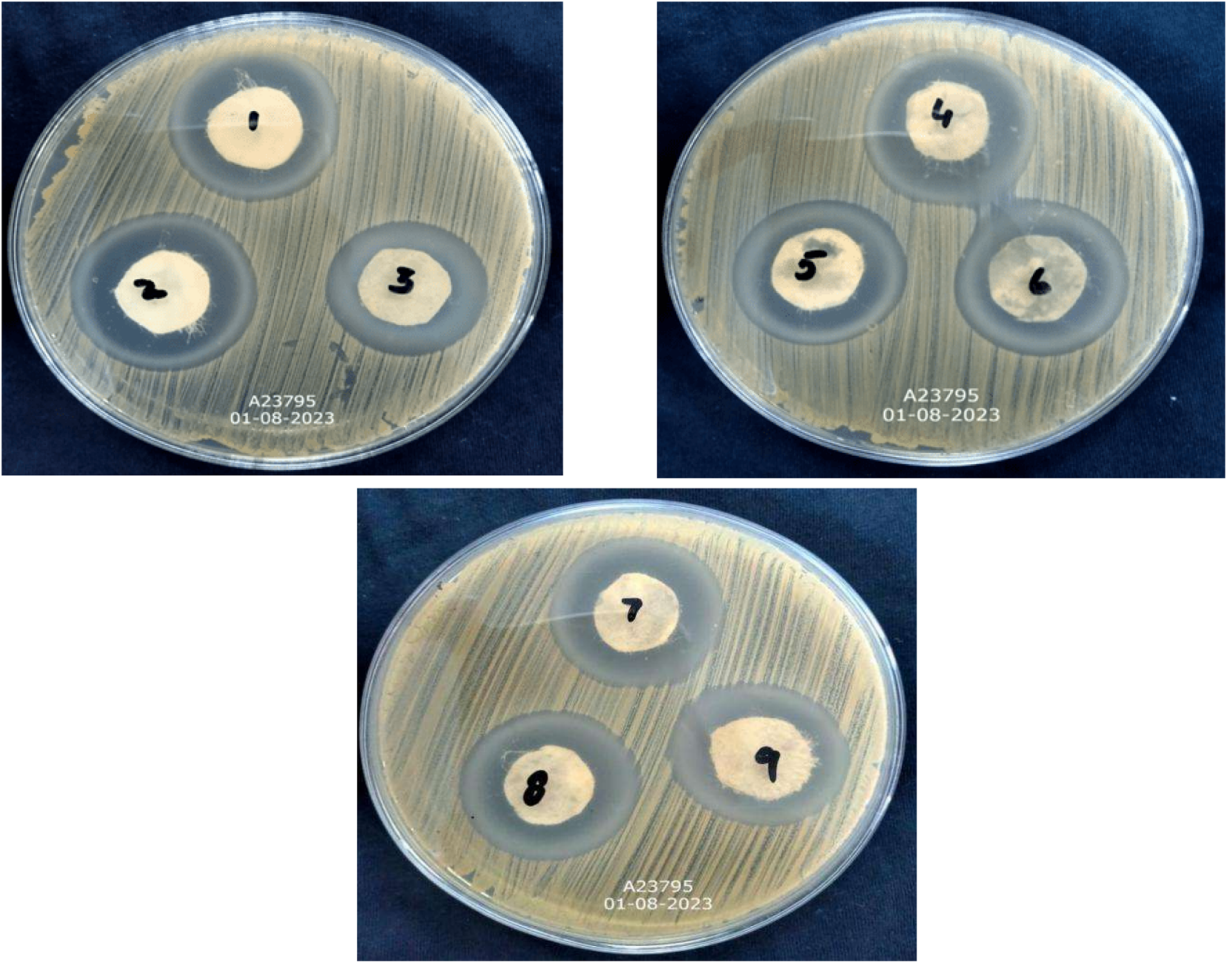
Antibacterial results of the hydrogel composite samples.

The hydrogel composite specimens, incorporating cotton nonwoven and coordinated with carboxymethyl cellulose hydrogel and silver nanoparticles, exhibited good antibacterial properties, as shown in Fig. 7. This was demonstrated by the development of inhibition zones around the composite specimens during testing, indicating adequate bacterial concealment. These outcomes were made possible by the addition of silver nanoparticles, which are well-known for their potent antimicrobial activity. Silver particles are known to disrupt bacterial cell layers, inhibit cellular respiration, and prevent bacterial DNA replication, ultimately leading to cell death. The changing sizes of the hindrance zones across the samples suggest a relationship with the hydrogel concentration and GSM. Silver ions are known to interact more effectively with the peptidoglycan layer of Gram-positive bacteria, which justifies their selection for the study. Silver nanoparticles are more likely to be present in samples with higher hydrogel and GSM concentrations, resulting in greater antibacterial activity. This upgraded action can be attributed to the increased surface area available for direct interaction with bacterial colonies. These results confirm that the hydrogel composites exhibit not only excellent exudate absorbency and moisture management but also significant antibacterial properties, making them promising candidates for wound-dressing applications.

## 4 Conclusion

A hydrogel composite was successfully created by incorporating cotton nonwoven fabric into the cellulose hydrogel and adding silver nanoparticles *via* a simple one-step approach. The composite's mechanical and functional characteristics, such as tensile strength, elongation, moisture control, and fluid absorption, were thoroughly examined. The results showed that increasing the hydrogel and GSM concentrations in the fabric improves mechanical properties and absorption, while decreasing elongation. Moisture management results showed successful wetting and spreading capacities, vital for maintaining a comfortable climate in wound applications. The composite also displayed prominent antibacterial properties, attributed to the presence of silver nanoparticles, which exhibited inhibition zones against bacterial growth. The results highlight the composite's practical capacity as a multifunctional material for wound care, with enhanced strength, moisture management, and antibacterial properties. The hydrogel composite created is a significant step forward in wound dressing design, as it improves patient outcomes and reduces the likelihood of infection.

## Conflicts of interest

There are no conflicts to declare.

## Data Availability

The datasets generated and analysed during the current study are available from the corresponding author on reasonable request.
